# The diverse roles of cytokinins in regulating leaf development

**DOI:** 10.1038/s41438-021-00558-3

**Published:** 2021-06-01

**Authors:** Wenqi Wu, Kang Du, Xiangyang Kang, Hairong Wei

**Affiliations:** 1 Beijing Advanced Innovation Center for Tree Breeding by Molecular Design, Beijing Forestry University, Beijing, PR China; 2 National Engineering Laboratory for Tree Breeding, Beijing Forestry University, Beijing, China; 3 Key Laboratory for Genetics and Breeding in Forest Trees and Ornamental Plants, Ministry of Education, College of Biological Sciences and Technology, Beijing Forestry University, Beijing, China; 4 College of Forest Resources and Environmental Science, Michigan Technological University, Houghton, MI USA

**Keywords:** Leaf development, Cytokinin

## Abstract

Leaves provide energy for plants, and consequently for animals, through photosynthesis. Despite their important functions, plant leaf developmental processes and their underlying mechanisms have not been well characterized. Here, we provide a holistic description of leaf developmental processes that is centered on cytokinins and their signaling functions. Cytokinins maintain the growth potential (pluripotency) of shoot apical meristems, which provide stem cells for the generation of leaf primordia during the initial stage of leaf formation; cytokinins and auxins, as well as their interaction, determine the phyllotaxis pattern. The activities of cytokinins in various regions of the leaf, especially at the margins, collectively determine the final leaf morphology (e.g., simple or compound). The area of a leaf is generally determined by the number and size of the cells in the leaf. Cytokinins promote cell division and increase cell expansion during the proliferation and expansion stages of leaf cell development, respectively. During leaf senescence, cytokinins reduce sugar accumulation, increase chlorophyll synthesis, and prolong the leaf photosynthetic period. We also briefly describe the roles of other hormones, including auxin and ethylene, during the whole leaf developmental process. In this study, we review the regulatory roles of cytokinins in various leaf developmental stages, with a focus on cytokinin metabolism and signal transduction processes, in order to shed light on the molecular mechanisms underlying leaf development.

## Introduction

In the late 1950s, a substance that promoted plant cell division was discovered in autoclaved herring sperm DNA and was called kinetin^1^. A few years later, a class of phytohormones with similar molecular structures, including 6-(γ,γ-dimethylallylamino)-purine, 6-benzyladenines, and zeatin, referred to as cytokinins^2^, were found to play important regulatory roles in cell division. Since then, the biosynthesis, metabolism, distribution, signaling pathways, and functions of cytokinins have been intensely investigated and characterized.

**Fig. 1:**
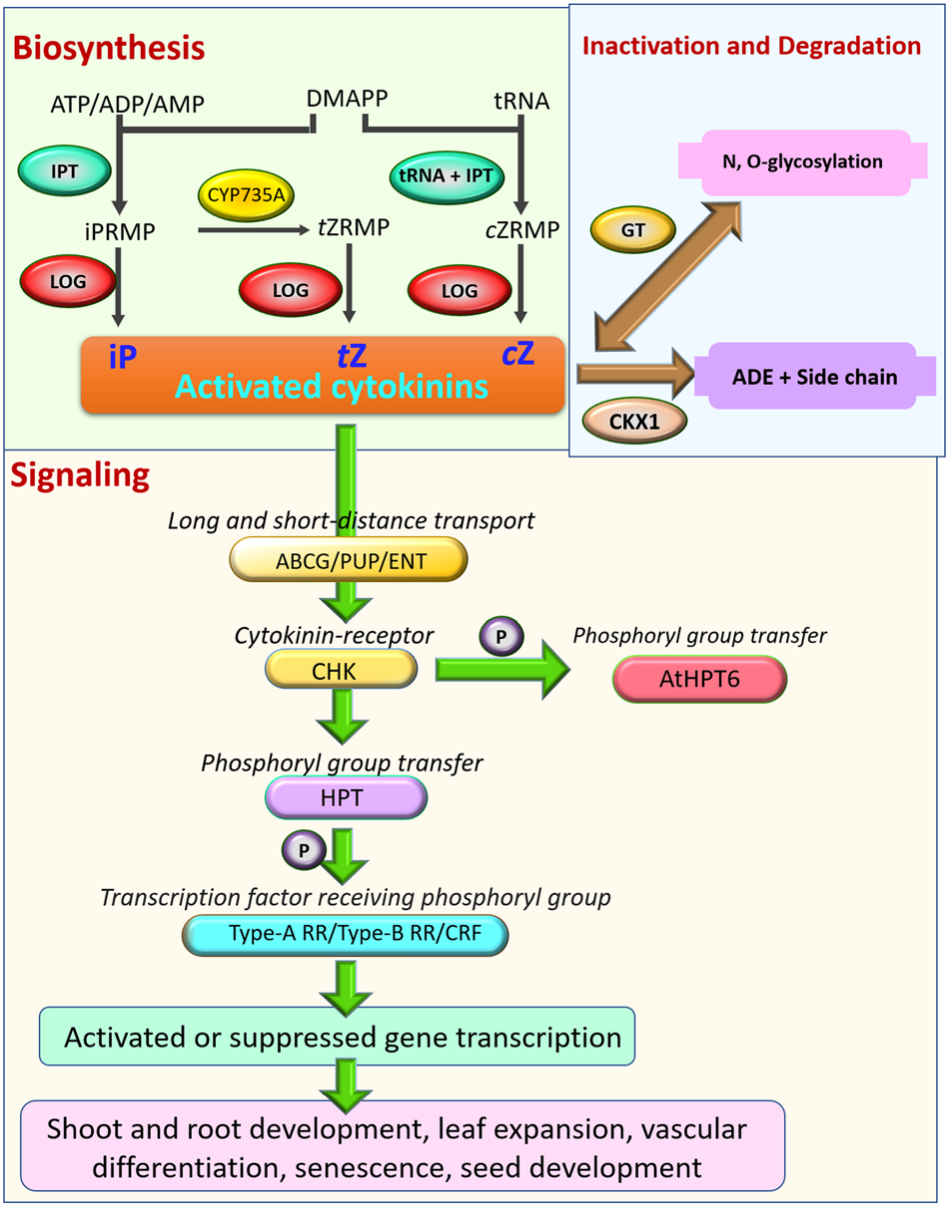
Schematic model of cytokinin (CK) biosynthesis, metabolism, degradation, and signal transduction. The names of the genes in up panels are shown in ovals, and in low panel are shown in the capsule shapes (see the text for further details). DMAPP: dimethylallyl pyrophosphate; iPRMP: isopentenyladenosine-5-monophosphate; *t*ZRMP, *trans*-zeatin riboside 5′-monophosphate; *c*ZRMP, *cis*-zeatin riboside 5′-monophosphate; iP, N6-(Δ2-isopentenyl)adenine; *t*Z: *trans*-zeatin; *c*Z: *cis*-zeatin; Ade: adenine; IPT, isopentenyltransferases; tRNA-IPT, tRNA-isopentenyltransferase; CYP735A, cytochrome P450 monooxygenase; LOG, LONELY GUY; GT, glycosyltransferase; CKX, cytokinin oxidase/dehydrogenase; ABCG, g subfamily ATP-binding cassette; PUP, purine permeases; ENT, equilibrative nucleoside transporters; HKs, histidine kinase; HPTs, histidine phosphotransfer proteins; ARR, response regulator, CRF, cytokinin response factor. Other abbreviations are as defined in the text

The main genes currently known to be involved in the cytokinin biosynthesis pathway encode the ISOPENTENYL TRANSFERASE (IPT) and LONELY GUY (LOG) enzymes^3,4^. The initial step of cytokinin biosynthesis in higher plants is the formation of cytokinin nucleotides, namely, isopentenyladenosine 5′-tri-, di-, or monophosphate (iPRTP, iPRDP, or iPRMP, respectively), from ATP, ADP, or AMP and dimethylallyl pyrophosphate (DMAPP) by IPTs^5^. *LOGs*, which encode phosphoribohydrolase-activating enzymes, directly convert a cytokinin nucleotide to an active free-base form of cytokinins in the final step of cytokinin biosynthesis^3^ (Fig. 1). The levels of active cytokinins can be modulated via irreversible cleavage by CYTOKININ OXIDASE (CKX) enzymes^6,7^ or through conjugation to glucose by cytokinin glycosyltransferases^8,9^ (Fig. 1). Plants regulate the concentration of active cytokinins through reversible and irreversible metabolism processes. Therefore, the precise maintenance of the homeostasis of cytokinins through these synthesis and inactivation enzymes is essential for plant development and adaptation to complex and changing environments. Recent studies have indicated that cytokinin biosynthesis varies with tissue and cell type^10,11^. However, as they are mobile signals, cytokinins rely on PURINE PERMEASES (PUP)^12,13^, EQUILIBRATIVE NUCLEOSIDE TRANSPORTERS (ENT)^12^, and G SUBFAMILY ATP-BINDING CASSETTE (ABCG)^14^ for short- and long-distance transport between roots and shoots (Fig. 1). In *Arabidopsis*, cytokinin signal transduction begins when cytokinins are received by sensor histidine kinases, HISTIDINE KINASE (HK2, HK3, and HK4), which initiate a phosphorylation signaling cascade in the endoplasmic reticulum^15,16^. After cytokinin binding, the phosphoryl group is transferred from HKs onto HISTIDINE-CONTAINING PHOSPHOTRANSMITTER (HPT) proteins^17^. HPTs then translocate from the cytoplasm to the nucleus and activate the transcription of *ARABIDOPSIS RESPONSE REGULATORS* (*ARRs*), which are categorized as type A transcriptional repressors^18–20^ or type B activators^20–22^, and *CYTOKININ RESPONSE FACTOR* (*CRF*)^20,23^ (Fig. 1). Through this signal transmission, cytokinins influence many aspects of biological processes that affect plant growth and development, such as cell division, apical dominance, shoot initiation and growth, phyllotaxis, vascular bundles, leaf senescence, branching and nodulation, seed germination, nutrient uptake, and biotic and abiotic stress responses^20,24,25^.

The development of plant leaves, which are the primary organs in plants for capturing light energy and perceiving diverse environmental conditions, is a dynamic process that can be divided into four different phases: the initiation of leaf primordia, the establishment of polarity (EP), the establishment of leaf size and morphology^26,27^, and leaf senescence^28^. First, cells at the peripheral zone of the shoot apical meristem (SAM) differentiate into a leaf primordium, whose position is regulated by phyllotactic patterning^29^. Second, the three growth axes, the adaxial–abaxial, proximal-distal, and mediolateral axes, are determined in the leaf primordium^30,31^. Even before the EP is completed, leaf primordium cells begin to divide and proliferate, which results in exponential increases in both leaf area and cell number. After the leaf blade and the petiole clearly separate, growth occurs throughout the entire leaf along the mediolateral axis, which results in the formation of the final shape of the leaf. After a growing season, flowering, nutrient deficiency, or unfavorable environmental conditions such as inadequate light or certain abiotic/biotic stresses, leaf senescence is initiated, which constitutes the final stage of the leaf lifespan preceding its death. However, these leaf developmental stages are not completely independent, as they are continuous and interconnected^26,27,29^.

In plants, cytokinins are essential regulators that are involved in almost every aspect of plant growth and development. During the various stages of leaf development, cytokinins play essential roles by regulating the transcriptional expression of downstream genes. Cytokinin homeostasis is modulated by certain transcription factors or by modulators during leaf development. Therefore, studies of the relationships among cytokinin signal transduction, gene regulation, and cytokinin modulation during various stages of leaf development help to reveal the underlying molecular mechanisms and advance our understanding in order to open novel avenues for improving agricultural and forestry yields. In this review, we focus on cytokinin homeostasis, signal transduction, and gene regulation as well as their regulatory roles in leaf development.

### The complex roles of cytokinins in leaf initiation

**Fig. 2:**
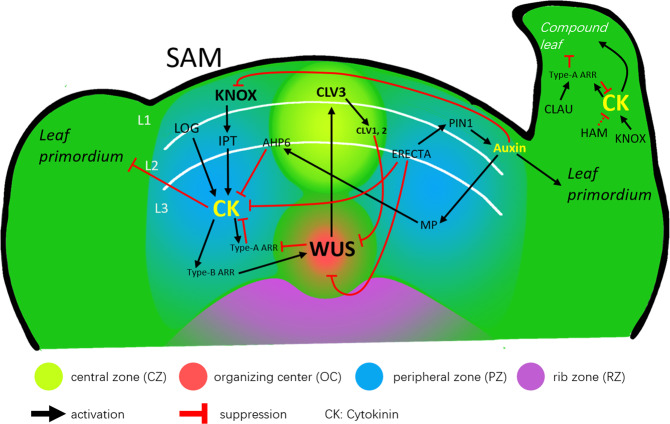
Schematic diagram of cytokinin (CK) regulation of leaf primordium initiation. Cells in the shoot apical meristem (SAM) are arranged into the L1, L2, and L3 layers and four distinct zones: the central zone (CZ), peripheral zone (PZ), organizing center (OC), and rib zone (RZ). KNOX is expressed in almost the entire SAM. KNOX positively regulates the synthesis of cytokinins and keeps their levels high. Cytokinins promote the expression of WUS through signal transduction and transcription factors, which maintain a high cell division rate in the OC. ERECTA blocks the effect of cytokinins and promotes the transport of auxin. In areas with higher auxin concentrations, the leaf primordium begins to form. In the early stage of leaf development, KNOX, which is highly expressed in the marginal blastozone, changes the leaf morphology by promoting cytokinin synthesis to form compound leaves. Solid lines indicate direct relationships that have been confirmed; dashed lines represent potential mechanisms. The abbreviations are as defined in the text

SAMs located at the shoot apexes are highly organized tissues containing pluripotent stem cells that can be divided into different functional zones, including the central zone (CZ), peripheral zone (PZ), and rib zone (RZ). SAMs generate nearly all the aerial organs and tissues of plants during postembryonic growth. Generally, cells in the CZ at the summit of the SAM divide slowly and maintain their pluripotency. The RZ below the CZ is responsible for generating stems. Some of the daughter cells produced by the CZ exhibit an accelerated cell division rate when they emerge in the PZ and eventually lead to the formation of lateral organs such as leaves and flowers (Fig. 2)^32,33^. Leaf primordia are initiated from the PZ, where cells become responsive to differentiation. Leaf primordia in the PZ are generated in a temporally and spatially controlled manner; this process is referred to as phyllotaxy^34^. The SAM is anatomically divided into three well-defined cell layers: The epidermal (L1) and subepidermal (L2) layers, known as the tunica, and an inner layer (L3) that is referred to as the corpus^32,35^. L1 and L2 are single-cell sheets with anticlinal cell division planes that form the epidermis of the plant tissue surface. The cells within L3 divide in all directions and form vascular tissues (Fig. 2)^26^. In response to plant hormones and external cues, the dynamic balance of cell division and differentiation can be perfectly controlled and maintained in the different subdomains of the SAM by cytokinin and auxin interactions as well as their homeostasis and spatial signaling.

Phytohormones, such as auxins and cytokinins, play indispensable but distinct roles during SAM development and maintenance. Auxins are required for leaf formation and organogenesis; in contrast, cytokinins promote meristem maintenance. However, these phytohormones do not exist and function independently; recent studies have shown that auxins and cytokinins function together in multiple cells, tissues, and organs with both antagonistic and synergistic actions^36–38^. The formation of new leaves in the apical meristem is initiated by the accumulation of auxin^36^. Unlike those of auxins, the primary functions of cytokinins in maintaining the size and structure of SAMs have been fully demonstrated in multiple experiments^25^. For instance, a reduction in the concentration of or sensitivity to cytokinins via mutation of *IPT*^39^, the overexpression of *CKX*^40^, or the modulation of signal transporter genes^41^ results in a decrease in SAM size and activity. Therefore, cytokinins play a central role in stimulating SAM activity and size through synergistic or antagonistic interactions with auxin. At the same time, many other regulators contribute to modulating cytokinin and auxin concentrations and gradients in different zones of the SAM.

Several regulators have been shown to play important roles in modulating the concentrations and activities of cytokinins. Transcription factor class I *KNOTTED-LIKE homeobox* (*KNOX I*) family genes^42^, including *SHOOT-MERISTEMLESS* (*STM*), *kn1-like in Arabidopsis thaliana1* (*KNAT1*), *KNAT2*, and *KNAT6*, are essential for establishing and maintaining meristem development by increasing cytokinin levels or sensitivity while simultaneously repressing GA. The expression patterns of *KNOX I* are primarily limited to the SAM, and *KNOX I* expression is absent in leaf primordia^43,44^. In *Arabidopsis*, STM activates the expression of *IPT7* in the SAM to promote cytokinin biosynthesis (Fig. 2). *stm* mutants, which exhibit shoot meristem loss, can be partially rescued through the application of exogenous cytokinins^45,46^. In addition, endogenous cytokinin overproduction significantly increases the mRNA levels of the *KNOX I* genes, which indicates that there may be a positive feedback loop between *KNOX I* genes and cytokinins in the SAM^47^. Another enzyme, *LOG4*, is expressed in the L1 layer and produces active cytokinins that move to the lower cell layer and form a diffusion gradient within the SAM^48,49^. Thus, *KNOX I* and *LOG4* provide a high level of cytokinin accumulation and activation in the SAM to sustain SAM growth and activity (Fig. 2).

The functions and effects of cytokinins in various cells in different zones are determined not only by their concentrations but also by their spatial signal transduction. Some genes regulate the size of the SAM by regulating the cytokinin signaling pathway to modulate the sensitivity of cytokinins. The main response genes involved in cytokinin signaling, *type-B ARRs* (*ARR1*, *10*, and *12*), directly bind and activate the homeodomain transcription factor *WUSCHEL* (*WUS*), which positively governs cell proliferation in the organizing center (OC) under a very high concentration of cytokinins^50,51^. In the peripheral area where *WUS* is expressed, multiple feedback systems mediated by hormonal components and transcription factors act in parallel to control the fate of meristems. WUS represses the *type-A ARRs ARR7* and *15* to positively strengthen cytokinin response sensitivity in the plant (Fig. 2)^52^. CLAVATA3 (CLV3), together with CLV1 and CLV2, forms a receptor complex that can also determine the size of the meristem by limiting the expression of *WUS*. In addition, WUS directly activates the expression of *CLV3* in the CZ, forming a negative-regulation feedback loop^53^. This local WUS–CLV3 feedback loop ensures a constant number of stem cells in the SAM (Fig. 2)^54,55^. Furthermore, a signaling pathway composed of ERECTA family receptors and epidermal characteristic factor-like ligands can limit the width of the SAM and promote leaf initiation by inhibiting the expression of *CLV3* and *WUS*^56,57^. Compared with wild-type *Arabidopsis* seedlings, mutant seedlings lacking all members of the ERECTA family are more sensitive to cytokinin treatment and exhibit increased SAM size and drastic changes in *WUS* and *CLV3* expression; this may occur because the buffer mechanism that maintains stem cell homeostasis against an increase in cytokinins is severely impaired^56^. Thus, the establishment of cytokinin homeostasis and the cytokinin gradient, as well as spatial signal transduction by cytokinins, play key roles in maintaining the structure of SAMs and their ability to continuously divide and grow.

The spatiotemporal pattern of lateral organ initiation at the SAM is controlled by both auxins and cytokinins^58^. Changing the auxin/cytokinin ratio requires additional feedback loops that stabilize phyllotaxis. During the initiation of leaf primordia, ERECTA family receptors not only inhibit the effect of cytokinins but also promote the formation of leaf primordia by increasing the expression of *PIN-FORMED 1* (*PIN1*), which increases polar auxin transport^59^. Auxins acting through MONOPTEROS (MP), an auxin-responsive transcription factor, activate the cytokinin signaling inhibitor *ARABIDOPSIS HISTIDINE PHOSPHOTRANSFER PROTEIN 6* (*AHP6*) to block the cytokinin signaling pathway (Fig. 2)^60^. AHP6 is not uniformly distributed in the SAM, resulting in different active regions of cytokinins within the SAM. This small spatial difference causes changes in the ratio of auxins to cytokinins that maintain the orderly production of leaf primordia^60,61^. However, studies have shown that cytokinins are a prerequisite for leaf initiation. Leaf initiation in tomato shoot apexes ceases in darkness but resumes under light or under cytokinin (zeatin) application to the summit of the meristem^62^. Cytokinins play two roles in this process: (1) promoting the growth of meristems to provide a source of stem cells as a prerequisite for leaf initiation and (2) affecting the establishment of the auxin gradient by regulating auxin biosynthesis and transport^62,63^. In monocotyledons, altered phyllotactic patterns are observed in the maize mutant *aberrant phyllotaxy1* (*abph1*)^64^ and the rice mutant *decussate* (*dec*)^65^. Both *ABPH1* and *DEC* encode proteins that function in cytokinin signaling, and the *abph1* and *dec* mutants have enlarged SAMs. However, mutants with disrupted cytokinin signal transduction pathways do not exhibit a phyllotactic shift^22^. In the *abph1* mutant, maize *PIN1* expression and auxins at the incipient leaf primordium are greatly reduced, as cytokinin specifically promotes the expression of maize *PIN1* in the incipient leaf primordium^66^. Collectively, these studies indicate that the phyllotactic shift may be a result of a delay in the initiation of lateral organs stemming from the change in the auxin/cytokinin ratio. In the initial stage of leaf development, the functions of cytokinins are contradictory; they delay the initiation of the leaf development process and suppress the formation of leaf primordia by inhibiting stem cell differentiation. However, cytokinins not only maintain the existence of the SAM but also provide cells for plant apical growth, which is a prerequisite for lateral organ formation and leaf primordium initiation.

### Cytokinins control leaf shape

The leaves of most plants have a flat and broad structure to support photosynthesis and gas exchange. Given their adaptations to different natural habitats, leaf forms can be broadly grouped into two categories: simple leaves, which consist of one entire unit with a single lamina, and compound leaves, which consist of multiple subunits called leaflets, each resembling a simple leaf^67^. Generally, the final shape of leaves has been shown to be determined by two biological processes: primary morphogenesis (PM), which determines the basic leaf form and structures, such as leaflets, lobes, and leaf margins, and secondary morphogenesis (SM), which includes most leaf expansion and differentiation and involves the production of cell types that are typical in mature leaves^33^. The development of a compound leaf requires the maintenance of temporospatial morphogenetic activity in the early stage of leaf development. For example, at the leaf margin, a region called the marginal blastozone (MB) is responsible for the organogenesis of structures such as lobes in simple leaves or leaflets in compound leaves^68^. In this case, the meristematic or stem cell identity in the MB or the marginal regions of leaflets needs to be maintained longer than that in the sinus regions to support compound leaf formation.

For prolonged activity in the MB and leaflet formation, cytokinins are involved in the maintenance of extended morphogenetic activity^69^. Increasing or decreasing endogenous cytokinin levels or readjusting cytokinin sensitivity in the developing leaf marginal meristem alters leaf complexity^70^. This change has been linked to the timely maintenance of morphogenetic capacity and regulation of cell proliferation by cytokinins along the margins of developing leaves^69,71^. During the formation of compound leaves, cytokinins also interact with auxins. The discrete distribution of the auxin response in the leaf margin is the key factor in the formation of compound leaves^70^. Both the local application of auxin in the developing leaf primordium and the increase in auxin sensitivity inhibit the supercompound leaf phenotype, which develops owing to an increased cytokinin concentration^69^.

In monocotyledons and dicotyledons, various mechanisms of compound leaf formation have been identified; some of them are common in both types of plants, whereas others have not been observed in dicotyledons. Previous studies have shown that the mechanisms regulating compound leaf development in dicotyledonous plant species, such as tomato, pea, *Cardamine*, and *Medicago*, are not entirely consistent. During simple leaf development, the downregulation of the *KNOX I* gene in leaf primordia is permanent. Unlike in the development process for simple leaves, *KNOX I* expression during compound leaf development is upregulated in the leaf primordium after leaf initiation, which leads to leaflet development; *KNOX I* expression eventually ceases, which leads to the acceleration of leaf maturation^72,73^. Overexpression or silencing *KNOX I* results in increased or decreased leaflet numbers in alfalfa^74^ and tomato plants^19,75^, respectively. During PM, KNOX I promotes cytokinin biosynthesis, similar to its effect in SAMs^69,76^. The reductions in cytokinin levels suppress the effect of *KNOX I* on leaf shape, and cytokinins can substitute for KNOX I activity at the leaf margin. Thus, cytokinins act downstream of KNOX I in the leaf margin (Fig. 2)^69^. Similar regulatory mechanisms that involve elevated cytokinins have also been shown to give rise to dissected or deeply lobed leaf morphogenesis in Araceae, a monocotyledonous family.

Increasing sensitivity to cytokinins has a similar effect on leaf morphology in dicots as elevating the level of cytokinins. One study has indicated that a change in the sensitivity of cytokinin signal transduction can affect leaf shape in tomato^77^. The CIN-TCP transcription factor family affects leaf shape by promoting differentiation. Overexpression of the CIN-TCP family gene *LANCEOLATE* (*LA*) in tomato leads to premature leaf differentiation and the production of smaller leaflets^77^. CIN-TCP in *Arabidopsis* inhibits cytokinin signal transduction and advances leaf cells to the expansion stage^78^. The decrease in MB activity in tomato may be mediated by the same mechanism of CIN-TCP regulation as in *Arabidopsis*. This mechanism has not been reported in *Cardamine*, pea, or *Medicago*. However, *UNIFOLIATA* (*UNI*)^79^ and *SINGLE LEAFLET1* (*SGL1*)^80^, which are homologs of *LEAFY* (*LFY*) in *Arabidopsis* that promote the cytokinin effect by inhibiting *type-A ARR7* expression^81^, have been reported to affect leaf shapes in pea and *Medicago*, respectively. *UNI* is expressed in the leaf blastozone and plays an active role in maintaining the blastozone. Both pea *uni*^79^ and *Medicago sgl1*^80^ mutants exhibited reduced leaf complexity because of the previously initiated differentiation of leaf cells. Therefore, UNI and SGL1 may regulate compound leaf development by altering cytokinin signaling. In tomato, other genes have been found to alter leaf morphology through cytokinin signaling. For example, the *CLAUSA* (*CLAU*) gene encodes an MYB transcription factor that regulates various aspects of tomato plant development. The tomato *clau* mutant is characterized by ectopic meristematic activity in leaves, which are highly complex and have many more secondary leaflets than wild-type tomato leaves^82^. Recent research has shown that CLAU attenuates cytokinin signaling by upregulating the expression of *type-A ARRs*, which negatively regulate the cytokinin response^71^. *CLAU* is of particular spatiotemporal relevance in compound leaves with regard to the cytokinin signaling pathway (Fig. 2). The *HAIRY MERISTEM* (*HAM*) gene encodes a GRAS family transcription factor that functions in meristem maintenance in compound leaf primordia^83,84^. In tomato, *ham* mutants exhibit overproliferation of meristematic cells in the compound leaf rachis; this phenomenon resembles the response to the elevation of cytokinin levels or sensitivity, leading to the proliferation of the ectopic shoot on the adaxial side of the compound leaf rachis^83^. The reduction in cytokinin levels in the *ham* mutant leaves completely suppresses the overproliferation phenotype. Thus, *HAM*, along with cytokinins, is required for the proper morphogenesis of the compound leaf (Fig. 2)^83^. In short, the final morphological development of leaves is likely accomplished through multiple concurrent regulatory mechanisms, and the temporospatial elevation of cytokinins and the altered sensitivities of their signal pathways in different leaf areas likely contribute greatly to compound leaf formation.

There are also other regulatory mechanisms related to compound leaf formation in monocotyledons. For instance, it has been reported that in the transition from cell division to cell expansion, the palm leaf primordium undergoes the second stage of leaflet separation, in which the number of cells on the folds of the ridge decreases. As a result, when the leaflets expand, mechanical force eventually separates them, resulting in the development of pinnate compound leaves^85^. In the other case, programmed cell death (PCD) has been suggested to play a role in the dissection of *Monstera* leaves; specifically, PCD causes perforations between two adjacent lateral veins during the early period of leaf expansion, and these perforations become enlarged as the leaf grows^86^. In brief, simple leaves subdivide into compound leaves through three mechanisms: (1) controlling marginal growth, which occurs in both dicotyledons and monocotyledons; (2) tissue abscission, which occurs in monocotyledons; and (3) PCD, which also occurs in monocotyledons. At present, cytokinins are known to be involved in controlling leaf marginal growth but not in leaf dissection through tissue abscission or PCD.

Recent studies have shown that cytokinins can affect the morphological development of simple leaves in monocotyledons. The leaf of a monocotyledon is composed of a leaf blade, ligule, auricle, and leaf sheath. In maize, the semidominant *HAIRY SHEATH FRAYED1* (*Hsf1*) mutation displays a mutant leaf phenotype that resembles the leaf pattern at the sheath-blade boundary, with outgrowths consisting of an organized auricle/ligule and a sheath emanating from the distal blade margin. Analysis of three independent *Hsf1* alleles revealed gain-of-function missense mutations in the maize cytokinin receptor *ZEA MAYS HISTIDINE KINASE1* (*ZmHK1*); the mutated residues near the cytokinin binding pocket enhance the cytokinin binding affinity and thus cause changes in the leaf pattern. Treating wild-type seedlings with exogenous cytokinins gives rise to the leaf phenotype of the *Hsf1* mutant^87^. Thus, cytokinins can influence the specifications of leaf patterning and alter leaf developmental programs in monocotyledons.

Among the various regulatory mechanisms that have been found to change leaf morphology, most increase the cytokinin concentration or cytokinin sensitivity to promote cell division activity in some leaf areas and ultimately result in dissected or altered leaf morphology in leaves. However, how these mechanisms function coordinately in through space and time and adapt to different environmental cues needs to be studied in greater detail.

### The relationship between cytokinins and leaf size

**Fig. 3:**
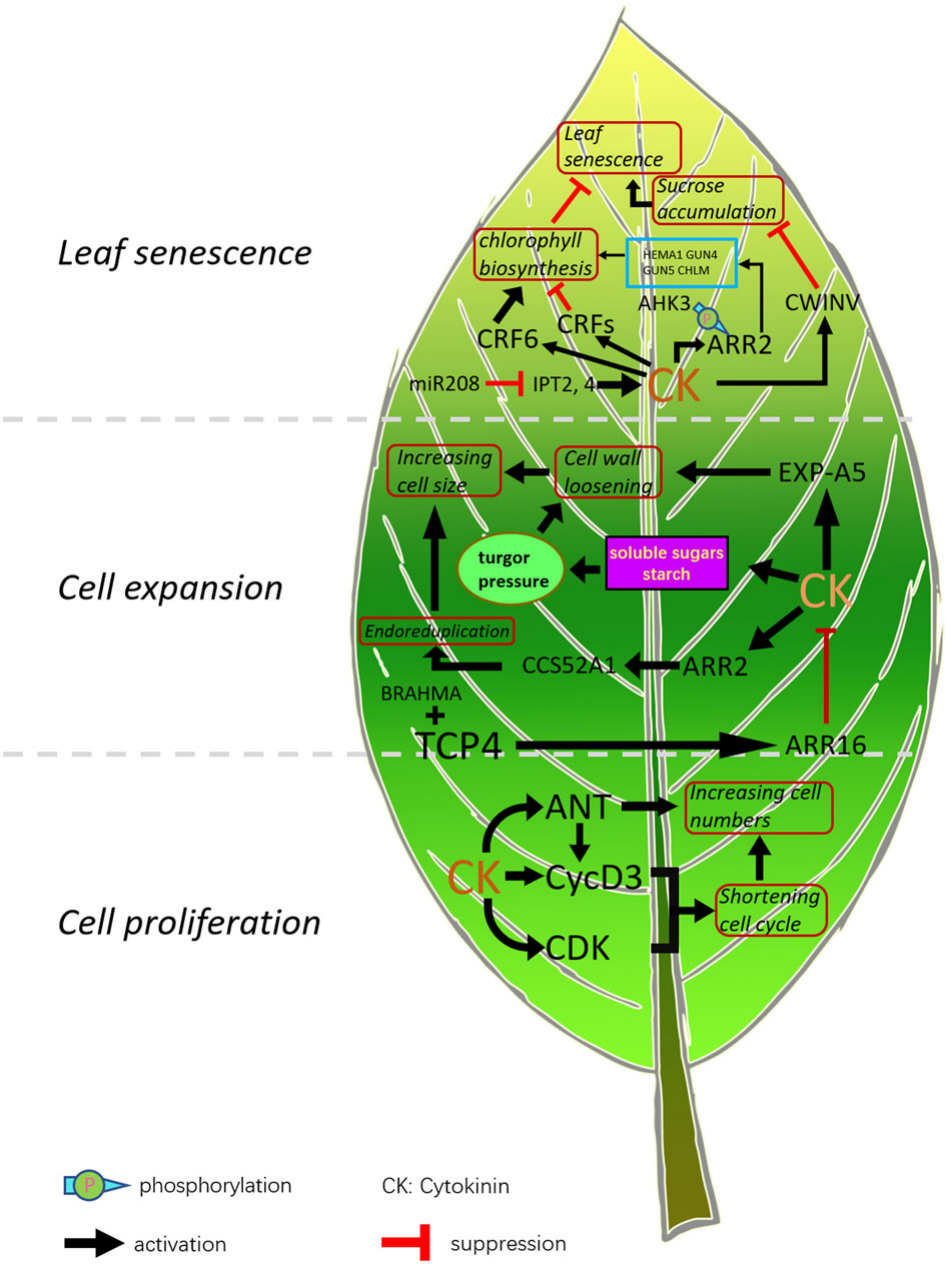
Schematic diagram of cytokinin functions in different stages of leaf development. The gray dashed lines divide the three stages of leaf development; the bright green area represents cells in the proliferation stage; the green area represents cells in the expansion stage; and the yellow-green area represents the senescence stage. The terms inside the red rounded rectangles are biological functions; the genes in the blue box are involved in chlorophyll synthesis. The solid arrows and blocked bars indicate activation and suppression, respectively

The size of a mature leaf is largely determined by the leaf cell numbers and cell sizes. After a leaf primordium emerges from the PZ as a rod-shaped protrusion at the flanking region of the SAM, all leaf cells undergo two biological processes, cell division and cell expansion, which determine cell numbers and cell sizes, respectively^88,89^. In addition, the timing of the transition between cell division and cell expansion indirectly affects leaf size^90^. In dicotyledonous plants, such as the model plant *Arabidopsis thaliana*, leaf growth is most often described as being influenced temporally by cell division. When the leaf primordium attains a certain size and position, leaf cell division begins to arrest at the distal tip of the leaf, which is termed the arrest front^88^. The arrest front boundary then moves downwards; when it reaches a certain point in the midleaf area, it stops for some time (generally a few days) before moving to the bottom of the leaf base^91,92^. After that, all leaf blade cells rapidly become committed to cell expansion^92^ (Fig. 3). In contrast, leaf development in monocotyledons is often seen as spatially regulated; that is, cell division occurs primarily at the base of a leaf, cell expansion in the middle of the leaf, and cell maturation at the tip of the leaf^93^. Therefore, the temporospatial distribution of cytokinins and their functional loci during leaf development may be quite different and determine the final leaf size.

Cytokinins control leaf size by regulating both leaf cell division and expansion; this has been known for many years^94–97^. During the leaf cell division phase, cytokinins, together with auxin, activate cell proliferation (Fig. 3)^98,99^. For instance, a cell culture study with cells in suspension showed that cell division arrests without auxin and that the addition of auxin to the arrested cell culture restores cell division activity^99^, suggesting that auxin provides the necessary signal that allows cells to enter the cell cycle^100–102^. However, cytokinins shorten the transition between any two adjacent cell cycle phases^103^ and extend the period of cell proliferation by delaying the onset of cell differentiation. Conversely, the onset of cell differentiation is brought about by cytokinin degradation caused by the upregulation of *CKX3*, which slows cell proliferation but expedites the onset of cell expansion^104,105^. After leaf development enters the cell expansion period, an excess of cytokinins stimulates cell expansion, resulting in plants with larger leaves that consist of larger cells, which leads to higher shoot biomass^104,106,107^. During leaf cell expansion, cytokinins are responsible for cell wall elongation^108^, turgor pressure increases^104^, and endoreduplication^109^. Endoreduplication, also known as endoreplication, gives rise to cells with extra copies of genomic DNA^110^ and contributes to the sizes of some specific types of cells in certain plant species during leaf expansion. Furthermore, auxin is also involved in cell expansion. Auxins are known to promote cell wall loosening and endoreduplication; this topic has been reviewed by Tsukaya^111^ and Perrot-Rechenmann^99^. In other tissues, such as roots, cell expansion is probably and mainly determined by the crosstalk between cytokinins and auxins^112^. However, how the temporospatial distribution and interaction of cytokinins and auxins change during the expansion phase of leaf cells remains largely unknown. In shaded environments, which significantly decrease the cytokinin contents in young, fully developed, and mature leaves, leaf size growth is impeded by a lack of cell expansion. Shaded environments reduce leaf expansion; however, when shaded leaves are treated with exogenous cytokinins, they achieve the same size as leaves grown under normal light conditions^113^. Defects in cytokinin signaling also result in reduced cell expansion. The sizes of the cotyledons of the triple *crf1,2,5* mutant are much smaller than those of the wild type, by nearly 96%; this is due primarily to the decline in cell expansion^114^. In conclusion, multifaceted cytokinin activities at distinct phases of leaf development shape the ultimate sizes of leaves. Recent studies have begun to unveil the underlying mechanisms by which cytokinins regulate cell division and expansion.

In the cell division phase, one of the mechanisms by which cytokinins control cell mitosis in leaf development is by modulating the expression of *D3-TYPE CYCLINS* (*CycD3*)^115^, *CYCLIN-DEPENDENT KINASES* (*CDKs*), and *AINTEGUMENTA* (*ANT*); CycD3, CDKs, and ANT encode a cell cycle regulatory protein^116^, serine/threonine kinases^103^, and a transcription factor^117^, respectively. During the cell proliferation stage of leaf development, cytokinins control cell division, which activates the G_1_/S and G_2_/M transitions in the cell cycle by promoting the expression of *CycD3*^98^ and *CDKs*^103^, respectively (Fig. 3). The more rapid transition from the G1 phase of the cell cycle to the S phase is crucial for the upregulation of eukaryotic cell proliferation^118^. CycD3 is a cell cycle regulatory protein that binds and activates *CDK*. The overexpression of *CycD3* is sufficient to induce cytokinin-independent shoot formation in the calli^98^, and the loss of CycD3 activity reduces the ability of exogenous cytokinins to induce shoot formation^115^. CycD3 promotes mitotic cell division and inhibits endoreduplication and cell differentiation. Therefore, CycD3 is considered the main means by which cytokinins interact with cell cycle control mechanisms. ANT is required for normal cell proliferation but not cell growth^119^. In situ hybridization experiments have shown that *ANT* mRNA accumulates in the primordia of all lateral bud organs. Shortly after the appearance of the primordium, *ANT* mRNA is localized in the growing regions of immature organs and functions^120^. The overexpression of *ANT* results in an increase in leaf and flower size, while loss-of-function *ant* mutants produce smaller leaves^119^. In response to exogenous cytokinins, *ANT* transcript levels increased relative to those in untreated plants (Fig. 3)^117^. Cytokinin deficiency in *CKX*-overexpression lines or in *ipt* mutants leads to a reduction in *ANT* transcript levels during root secondary growth or early leaf proliferation^104,117^. Some genes control leaf size by regulating cytokinin accumulation or signaling pathways to change the number of leaf cells and ultimately change the size of the leaves. *GROWTH-REGULATING FACTORs* (*GRFs*) are a gene family of transcription factors that regulate various aspects of plant growth and development. In a variety of plant species, the overexpression of most *GRF* genes leads to the enlargement of lateral organs. In *A. thaliana*, the number of leaf cells in transgenic plants with *GRF5* overexpression increased, and the leaves became larger. The expression of *CRF2*, which is a gene downstream of GRF5, is significantly upregulated, and the corresponding sensitivity of cytokinins is increased^121^. In poplar, GRF5-1 can bind to the promoter of *CKX1* and inhibit its expression. The cytokinin concentration in the apical buds and immature leaves of *GRF5-1*-overexpressing transgenic plants increased, which increased the number of mesophyll cells and leaf area^122^. In brief, during the leaf cell proliferation stage, cytokinins increase the number of leaf cells by promoting the mitotic replication cycle and accelerating cell division in different facets, thereby regulating leaf size.

Cytokinins are also involved in the cellular state transition from cell division to cell expansion. The TEOSINTE BRANCHED1/CYCLOIDEA/PCF transcription factor family (TCP), which targets growth-related genes, is composed of two classes that antagonistically control leaf growth in a spatially restricted manner^78,123^. TCP4, belonged to class II proteins (also known as CIN-TCPs), promotes the change from leaf cell division to leaf expansion by activating cell differentiation and accelerating the progression of the cell cycle arrest front^78,107^. CIN-TCPs recruit BRAHMA, a component of the SWI/SNF chromatin remodeling complex, to bind the promoter of *ARABIDOPSIS RESPONSE REGULATOR16* (*ARR16*), which is a type-A negative regulator of cytokinin response, and activate the expression of *ARR16*^107^. Thus, the reduction in sensitivity to cytokinins due to the expression of *ARR16* is thought to be associated with differentiation in leaf growth (Fig. 3). CIN-TCP genes control cell division arrest in the early stage of leaf development and thereby ensure that the leaves remain flat. A reduction in CIN-TCPs expression results in delayed basal node progression and increased cell proliferation before ultimately blocking mitosis^78^. In short, the blockage of cytokinin signaling is critical to the formation of the arrest front boundary.

In the cell expansion phase, cytokinins are shown to be involved in at least three mechanisms that contribute to the final cell size. During this phase, cell walls undergo loosening, remodeling, and biosynthesis^124^. Growing plant cells characteristically exhibit faster cell elongation under acidic conditions, which are induced by auxin through the stimulation of plasma membrane H^+^-ATPase proton pump activity^125,126^. Expansins are cell wall proteins that induce pH-dependent wall extension and stress relaxation and comprise a large superfamily with at least two major branches (identified as α-expansins (EXPA) and β-expansins (EXPB))^127^. The interplay between cytokinins and expansins in cell growth has been reported in a few plant species, such as *Arabidopsis*^128^, *Melilotus*^108^, soybean^129^, *Rosa*^130^, and poplar^131^. In poplar trees, the highest levels of *EXPA3* mRNA are observed in young leaves that will expand in size. Furthermore, the expression of *EXPA3* is inducible by exogenous cytokinins^131^. In addition, a recent study showed that *CKX2* activation induces the expression of *EXPA5* within 3 h (Fig. 3)^104^. In *Arabidopsis*, constitutive overexpression of CKX1 leads to a decrease in the number of leaf cells but an increase in leaf cell size^40^; these responses might be a compensatory mechanism related to the enhancement of postmitotic cell expansion in response to a decrease in cell number during lateral organ development^132^. In the root growth zone of *Arabidopsis*, both cytokinins and auxins can induce the expression of certain expansin genes^133^. Presumably, this is true in leaves as well, and the underlying activation mechanisms are worth investigating. Proteome profiling of leaves with excessive cytokinin during the cell expansion phase revealed that carbohydrate metabolism and energy-associated processes were stimulated. These processes result in significantly increased contents of major soluble sugars and starch in response to an excess of cytokinins^104^, which increases turgor pressure and is required for the biochemical loosening of the cell wall (Fig. 3)^127^. Endoreduplication, the increase in ploidy by chromosome replication without subsequent cell division, is also often involved in the process of increasing cell sizes^134^. In *Arabidopsis*, type-B ARR2 binds to and activates the *CCS52A1* gene (null function alleles of which reduce endoreduplication expansion in leaf cells^109^) and promotes the onset of the endocycle^135^. In cytokinin receptor mutants, *CCS52A1* expression was reduced^136^; consequently, reduced cell endoreduplication was observed^135^ (Fig. 3). In summary, cytokinins increase cell size in plants by promoting cell wall elongation, increasing turgor pressure, and enhancing endoreduplication.

In conclusion, cytokinins regulate the rate of cell division, the time of transition, and the extent of cell expansion, thereby affecting the numbers and sizes of cells and eventually the leaf size. However, the modes of action of cytokinins during the above processes remain largely unclear or unknown, and further research should focus on the molecular and biochemical mechanisms underlying these processes.

### Cytokinins delay leaf senescence

The final stage of leaf development is senescence, which can significantly affect the survival, health, and productivity of plants during the growing season. Senescence is characterized by color changes in both perennial and annual plants in the late summer and throughout autumn. In this phase, the most perceptible phenotypic change that embodies senescence is the appearance of variegated leaves, which develop due to the disassembly of chloroplasts and the degradation of proteins, lipids, nucleic acids, and pigments^111,137^. The nutrients that are generated from the degradation of senescing leaves are transported to developing seeds and fruits in annual plants or to new leaves or flowers in perennial trees, resulting in the death of the senescing leaves^137,138^. Therefore, although leaf senescence is an adverse process for leaf organs, it represents an altruistic death that plays a vital role in maintaining plant adaptability by ensuring the production of fit offspring and improving plant survival with a given spatiotemporal ecological niche^139^. Leaf senescence is influenced by various endogenous signals (plant hormones and age) and environmental signals (darkness, shading by other plants, UV-B or ozone exposure, nutrient limitation, extreme temperatures, drought, high salinity, and pathogen attacks)^137,140,141^. Under most environmental conditions, leaf senescence is initiated and develops due primarily to leaf age. Various plants shed their old leaves at different times during the growing season. Abiotic and biotic stresses can also enhance this process and may reduce plant biomass accumulation.

Leaf senescence is not a passive, unregulated degeneration process. Phytohormones, especially cytokinins^142^ and ethylene^143^, have been reported to affect leaf senescence; specifically, cytokinins are thought to delay leaf senescence, whereas ethylene is thought to induce it. Cytokinins are believed to serve as negative regulators of leaf senescence in a variety of monocotyledonous^144–147^ and dicotyledonous^148–151^ species. A reduction in cytokinin levels before the onset of senescence is believed to be a key signal for the initiation of senescence^142^. The exogenous application of cytokinins or the transgenic expression of cytokinin biosynthesis genes prevents the degradation of chlorophyll, photosynthetic proteins, and RNA, resulting in delayed senescence (Fig. 3)^142,147,152,153^. For example, transgenic tobacco with a senescence-specific gene (*SAG12*) promoter fused with the *IPT* gene significantly impedes leaf shedding and other symptoms of senescence^142^. Moreover, because of the prolongation of photosynthetic activity, the biomass of the transgenic plants and their seed productivity were greatly augmented. In tomato, overexpression of the *IPT* gene (*pSAG12::IPT* and *pSAG13::IPT*) inhibits leaf senescence, promotes earlier flowering, and increases fruit weight and total soluble solids^154^. The increase in biomass accumulation is to a large extent due to the extended photosynthetic period and nutrient transport in senescent leaves. In contrast, cytokinin biosynthesis mutants have a shorter leaf life span^148,155^. miRNAs are important posttranscriptional regulators of plant growth and development that participate in the process of leaf senescence by modulating cytokinins. Recent studies have shown that a newly identified microRNA (*miR208*) in tomato that reduces cytokinin biosynthesis by regulating *IPT2* and *IPT4* post-transcriptionally promotes leaf senescence (Fig. 3)^148^. Cytokinins can postpone leaf aging processes caused by unfavorable or adverse environmental conditions (such as drought or darkness) and delay the occurrence of leaf senescence^146,156^. Various experiments on different genes in many plant species have consistently shown that the levels of cytokinins play a major role in both single-leaf and whole-plant senescence.

Although the senescence-delaying effects of cytokinins have been well established, the mechanisms behind this phenomenon remain largely unknown. However, recent research on cytokinin signal transduction has shed new light on the underlying mechanisms. Cytokinin signal transduction genes such as *AHK3*, *type-B ARR2*, and *CRFs* are closely associated with the development of leaf senescence^157,158^. In *Arabidopsis*, the overexpression of either *AHK3* or *ARR2* leads to a delay in leaf senescence. However, the overexpression of *ARR2* without the AHK3 phosphorylation site does not lead to phenotypic changes. This result suggests that AHK3 plays a major role in controlling cytokinin-mediated leaf longevity through the specific phosphorylation of the response regulator ARR2 (Fig. 3)^157^. Additional research has shown that in the *akh2,3* double mutant, the expression levels of chlorophyll synthesis genes *HEMA1*, *GUN4*, *GUN5*, and *CHLM* were reduced, which suggests that cytokinins can increase chlorophyll content and delay leaf senescence (Fig. 3)^159^. *CRFs*, which encode cytokinin signal transduction transcription factor family genes, are induced by cytokinins and exhibit different biological functions during the process of leaf senescence regulation. In dark-induced excised-leaf assays, leaves overexpressing *CRF6* retain more chlorophyll than the wild type leaves without exogenous cytokinins, indicating that CRF6 negatively regulates leaf senescence^158^. However, the growth phenotype of the *crf6* mutant line did not differ from that of the wild type, and no premature senescence was observed. In contrast, leaves of *CRF1-*, *CRF3-*, and *CRF5*-overexpressing transgenic lines developed senescence earlier than the wild type leaves, while the *crf1,3,5,6* and *crf1/CRF1,2,5,6* mutants exhibited delayed leaf senescence^160^. These results may be caused by differences in the regulation of plant development by cytokinins in different environments. Sucrose accumulation is an important signal for the induction of leaf senescence^137^. During plant development, young leaves (a substantial sink) need the hexoses provided by old leaves (a source) as energy until the young leaves are mature enough to supply themselves with energy through photosynthesis. After the demand from other organs decreases, sucrose then gradually accumulates in the old leaves and induces senescence^140^. Cytokinins have long been shown to accelerate nutrient mobilization during the establishment of new source-sink relationships^161^. Cytokinins are known to increase the activity of cell wall invertase (CWINV), which plays a key role in regulating the source-sink relationship and is considered one of the main modulators of sink activity^162–165^. The CWINV enzyme catalyzes the cleavage of sucrose into hexose monomers on the cell wall and transports them to sink organs. The increase in CWINV activity reduces the accumulation of sucrose in old leaves, which delays leaf senescence. Restricting the activity of CWINV has been shown to block the function of cytokinins in inhibiting leaf senescence^162^. However, the interrelationship between cytokinins and CWINV with regard to leaf senescence can be complicated by other factors; thus, more experiments are needed to characterize their effects on leaf senescence. In conclusion, cytokinin signal transduction genes delay the biological process of leaf senescence by increasing the chlorophyll content and reducing sucrose accumulation in leaves.

### Concluding remarks

Given the urgent demand for renewable energy and the increased interest in bioenergy, understanding the biochemical and molecular mechanisms underlying leaf development has become increasingly important; such studies can help scientists find a way to increase plant growth and biomass in various agricultural crops and woody plant species. In this review, we summarized the roles of cytokinins in various leaf developmental stages. At the initial stage of leaf development, cytokinins sustain the growth of SAMs and provide sufficient stem cells for the leaf primordium to protrude. Cytokinins then regulate the synthesis and transport of auxin to promote the emergence of leaf primordia. At later stages, cytokinins promote cell proliferation, which increases the number of leaf cells in a short period and represses the transition of leaf cells into the expansion stage. The development of leaves from simple to compound morphology is a complicated process that is still not fully understood. Nevertheless, it is clear that cytokinins play a role in this process by maintaining the activity of leaf margin meristems. After leaf cells develop and grow into the expansion stage, cytokinins promote cell expansion by promoting cell wall elongation, increasing turgor pressure, and enhancing endoreduplication. In the final stage of leaf development, cytokinins maintain chlorophyll synthesis while slowing the process of leaf senescence. Thus, cytokinins play an important role during the entire process of leaf development. However, as most of the underlying molecular mechanisms remain unclear, there is a need for more work to be done to advance our understanding of these mechanisms. Cytokinins shorten the cell replication cycle by activating the transcription of *CycD3*, but little is known about the factors that mediate between cytokinins and *CycD3*. *ARR* and *CRF* genes, which can activate or inhibit the transcription of downstream genes, belong to large gene families, and which of these genes are regulated by these transcription factors at different stages of leaf development remains to be determined. Excessive cytokinins can change the leaf morphology of both monocotyledons and dicotyledons, but whether the leaves of these two types of plants are regulated by the same molecular mechanism requires further study. Plant hormones such as cytokinin, auxin, ethylene, brassinosteroid, gibberellins, abscisic acid, jasmonic acid, and others interact with each other and affect all stages of leaf development. Understanding the relationships between the ratio of these hormones and leaf development will benefit plant tissue culture and provide new insights into plant development. Therefore, more focused studies on the mechanisms of action of cytokinins and their synergistic interactions with other hormones are needed to advance our understanding of leaf development.
